# Mitochondrial Genomes of Three *Melampus* Species (Ellobiidae; Gastropoda)

**DOI:** 10.1002/ece3.71282

**Published:** 2025-04-11

**Authors:** Thomas Inäbnit, Ralph Tiedemann, Alice B. Dennis

**Affiliations:** ^1^ Unit of Evolutionary Biology/Systematic Zoology Institute of Biochemistry and Biology, University of Potsdam Potsdam Germany; ^2^ Laboratory of Adaptive Evolution & Genomics, URBE, Institute of Life, Earth and Environment (ILEE), University of Namur Namur Belgium

**Keywords:** control region, gastropod, *Melampus*, mitochondrial genomes

## Abstract

*Melampus bidentatus* was recently divided into three genetically divergent cryptic species: *Melampus bidentatus* , *Melampus jaumei*, and *Melampus gundlachi* . We have assembled and annotated a mitochondrial genome for each of these species. Comparisons with other taxa showed that these three species possess a gene order that is distinct from the conserved gene order found in the six (out of nine available) Ellobiid genera. Among the species with a derived gene order, mean nucleotide divergence over all genes was on average 1.57× higher than among mitogenomes with the conserved gene order. This suggests varying evolutionary rates of mitochondrial genes in this family.

## Graphical abstract

The mitochondrial genomes of three gastropod species from the family Ellobiidae— *Melampus bidentatus* , *Melampus jaumei*, and *Melampus gundlachi*—were assembled and annotated and found to have a gene order that diverges from the conserved gene order that is recovered in most genera within Ellobiidae and—more generally—within Euthyneura. Mitochondrial genomes within Ellobiidae that differ from the conserved gene order were found to be more divergent compared to each other than the mitochondrial genomes that have the conserved gene order, indicating variable evolutionary rates of mitochondrial genes within the family. Comparisons among 130 mitochondrial genomes within Euthyneura supported the presence of a control region preceding the isoleucine‐tRNA, including in the novel genomes presented here.

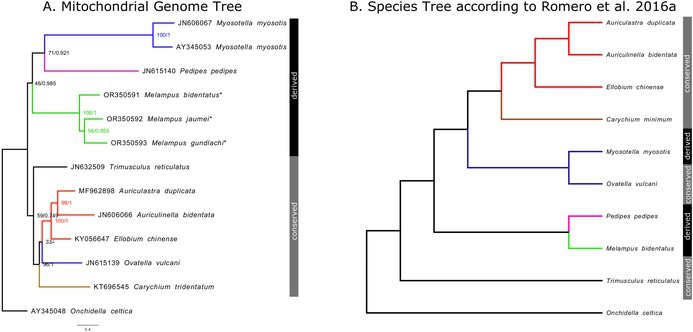

## 1 Introduction

**FIGURE 1:**
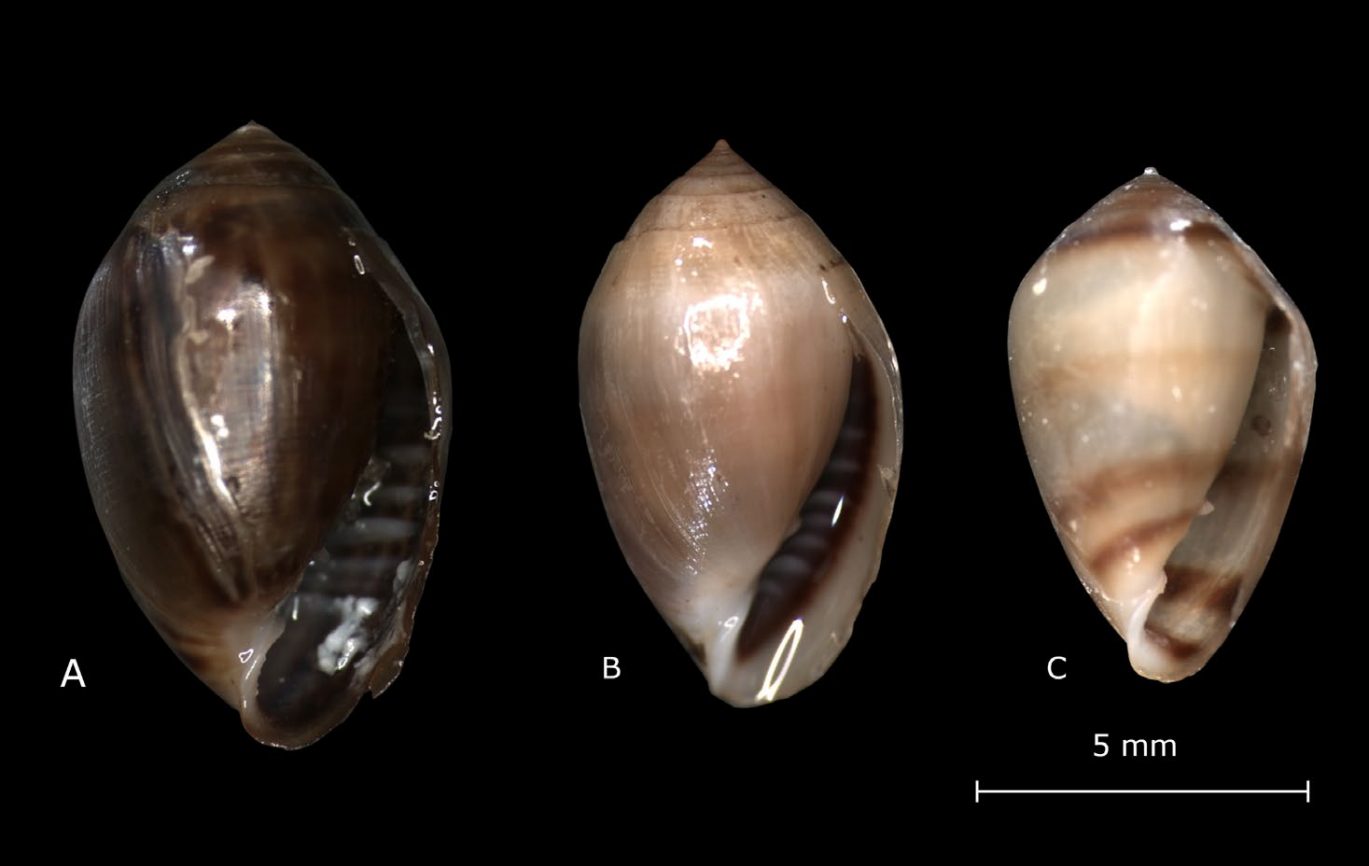
Images of the shells of three *Melampus* species sequenced in this paper. (A) *Melampus bidentatus* from Beebe Cove, Connecticut; (B) *Melampus jaumei*, a specimen from the same collection site (Dauphin Island, Mobile Bay, Alabama) as the sequenced specimen; (C) *Melampus gundlachi* from the same collection site (South Padre Island, Texas) as the sequenced specimen. Photos taken by Alice Dennis.

The coffee‐bean snails *Melampus bidentatus* Say 1822, *Melampus jaumei* Mittre 1841, and *Melampus gundlachi* Pfeiffer 1853 are cryptic air‐breathing Ellobiids that were until recently considered conspecific (Dennis and Hellberg 2010; Inäbnit et al. 2023; Figure 1). They are distributed in distinct ranges: *M. bidentatus* occurs from New Brunswick to Florida, *M. jaumei* in the northern Gulf of Mexico, Atlantic Florida, and from North Carolina to Delaware, and *M. gundlachi* in southern Florida, southern Texas, and the Greater Antilles (Dennis and Hellberg 2010). The ranges are parapatric, with *M. bidentatus* and *M. jaumei* co‐occurring between North Carolina and Delaware.

Analysis of COI sequences has shown high divergence between the species (22%–24%; Dennis and Hellberg 2010), but other mitochondrial genes have not been analyzed. In other Ellobiid species, phylogenies constructed from whole mitogenomes (e.g., Varney et al. 2021) have recovered species that were assigned to the family based on other genetic markers (Romero, Pfenninger, et al. 2016) and morphology (Martins 1995, 1996, 2007) outside the family (e.g., *Myosotella myosotis* , *Pedipes pedipes*). These Ellobiids possess distinct mitochondrial gene orders relative to the conserved gene order within Euthyneura (i.e., Heterobranchia without “lower” taxa: White et al. 2011; Varney et al. 2021) and most Ellobiids. This study aims to unravel the divergence across entire mitogenomes in the formerly cryptic *Melampus* species and to place it in context with the mitogenome architecture across Euthyneura.

## 2 Material and Methods

### 2.1 Collections and Sequencing

The mitochondrial genomes of three species were assembled in this study, each collected in sites of allopatric occurrence (Table 1). These collections did not require any special permission or permitting. Specimens were preserved in EtOH (> 95%) and stored at 20°C prior to extraction.

**TABLE 1:** Collection sites for the three species for which mitochondrial genomes are presented here.

Species	Collection site	Collection date	Coverage
*Melampus bidentatus*	Jamestown, Rhode Island (Latitude: 41.507, Longitude: −71.375)	22 November 2022	621.226×
*Melampus jaumei*	Mobile Bay, Alabama (Lat.: 30.251, Long.: −88.201)	8 December 2020	1253.09×
*Melampus gundlachi*	South Padre Island, Texas, USA (Lat.: 26.139, Long.: −97.176)	16 May 2006	265.34×

For *M. jaumei*, DNA was extracted using a modified 2× CTAB protocol. Briefly, chopped tissue was digested in 600 μL of a 2× CTAB buffer (1.4 M NaCl, 20 mM EDTA, 100 mM Tris–HCl pH 8.0, 2% CTAB, and 2% SDS), mixed with 200 μg proteinase K and 2 μL 2‐mercaptoethanol. Samples were incubated overnight at 55°C, with a second additional 200 μg of proteinase K added after approx. 8 h. Digested samples were mixed with 600 μL of cold phenol‐chloroform‐isoamyl alcohol (25:24:1) and centrifuged at max speed for 10 min at 4°C. The aqueous layer from this was isolated and mixed with 600 μL cold chloroform. Finally, the aqueous layer from this was isolated and DNA was precipitated with 600 μL cold isopropyl alcohol, washed twice with 75% EtOH, and eluted in 100 μL HPLC pure water. Extracted DNA was sent to Novogene (Cambridge, UK) for library preparation and PacBio HiFi sequencing.

*Melampus bidentatus* was extracted using the DNeasy Blood & Tissue kit (Quiagen 69504) tissue protocol according to the manufacturer's instructions and including an overnight tissue digestion. *Melampus gundlachi* DNA was extracted using the MagAttract HMW kit (Qiagen 67536) tissue protocol, and included overnight tissue digestion. For *M. bidentatus* and *M. gundlachi* , DNA was sent to the University of Leiden for library preparation (ultra‐low input and low‐input sequencing library preparation, respectively) and PacBio HiFi sequencing.

### 2.2 Assembly and Annotation

Mitogenomes of *M. jaumei* and *M. gundlachi* were assembled from PacBio Hifi data using MitoFinder v1.4 (Allio et al. 2020) with standard settings. MitoHiFi v2.0 (Uliano‐Silva et al. 2022) was then used to extract and circularize the resulting mitochondrial genome sequence. To get the mitogenome of *M. bidentatus* , the Hifi reads were mapped to the *M. jaumei* mitogenome with minimap2 v2.28 (Li and Durbin 2009, 2010; Li 2017). The mapped sequences were then extracted using samtools v1.19.2 (Danecek et al. 2021) and assembled using hifiasm v0.20.0 (Cheng et al. 2021). Minimap2 and hifiasm were run on the Galaxy Europe server (https://usegalaxy.eu; The Galaxy Community 2024). Annotation for *M. jaumei* and *M. gundlachi* was performed using the online MITOS webserver (Bernt et al. 2013); annotation for *M. bidentatus* was performed using MITOS2 v2.1.9 (Arab et al. 2017; Donath et al. 2019), as implemented on Galaxy Europe. In both cases, the “Invertebrate Mitochondrial” genetic code was used. Annotations were manually checked for the presence of start and stop codons and adjusted if protein‐coding genes did not start on start codons or stop on stop codons. Features that were not annotated by MITOS were manually added using comparisons with other *Melampus* mitochondrial genomes where those features were annotated. Average coverage was estimated using samtools stats with a bam file created by mapping the Hifi reads to the respective mitogenome using minimap2.

In order to add to the ongoing (Grande et al. 2008; White et al. 2011; Kurabayashi and Ueshima 2000) discussion on the position of a putative control region, all non‐coding regions from complete mitochondrial genomes, both produced here and from further Ellobiidae available on GenBank (Table 2, as of the time of submission) were aligned and compared. Specifically, we searched for non‐coding regions in the same positions in all of these mitochondrial genomes. The presence of those non‐coding regions was further evaluated in 151 Heterobranch (mostly Euthyneuran) mitochondrial genomes downloaded from GenBank (Table S2).

**TABLE 2:** Mitochondrial genomes from Ellobiidae used in our analyses.

Species	GenBank accession number	Reference	Gene order
*Auriculastra duplicata*	MF962898	Yi et al. (2017)	Conserved
*Auriculinella bidentata*	JN606066	White et al. (2011)	Conserved
*Carychium tridentatum*	KT696545	Romero, Weigand, et al. (2016)	Conserved
*Ellobium chinense*	KY056647	Jun et al. (2016)	Conserved
*Melampus bidentatus*	OR350591	This study	Distinct
*Melampus gundlachi*	OR350593	This study	Distinct
*Melampus jaumei*	OR350592	This study	Distinct
*Myosotella myosotis*	AY345053	Grande et al. (2008)	Distinct
*Myosotella myosotis*	JN606067	White et al. (2011)	Distinct
*Ovatella vulcani*	JN615139	White et al. (2011)	Conserved
*Pedipes pedipes*	JN615140	White et al. (2011)	Distinct
*Trimusculus reticulatus*	JN632509	White et al. (2011)	Conserved

All coding regions of all available mitochondrial genomes within Ellobiidae (Table 2; as of time of submission) were aligned using ClustalW as implemented in Mega X (Tamura et al. 2004; Kumar et al. 2018). Two separate subsets were created, containing (1) species with the conserved gene order of Euthyneura that are consistently recovered as monophyletic in mitochondrial genome‐based phylogenies (e.g., White et al. 2011; Varney et al. 2021) and (2) species with derived gene orders that are consistently recovered outside the first subset in mitochondrial genome‐based phylogenies (e.g., White et al. 2011; Varney et al. 2021) but are consistently recovered within the first subset in phylogenies that include nuclear markers (Romero, Pfenninger, et al. 2016) and in morphological studies (Martins 1995, 1996, 2007). In order to try to find out why the second subset is recovered outside the first subset in phylogenies based on mitochondrial genomes, the overall mean distances were calculated for both subsets for each coding region and the amino acid sequence of all protein‐coding genes, as well as for the full alignments of the three *Melampus* mitogenomes and the Ellobiids with conserved gene order in Mega X. For the three *Melampus* species, pairwise distances were calculated in Mega X for all genes separately and the mitochondrial genome as a whole.

Phylogenetic trees were calculated using the protein‐coding genes of all published Ellobiid mitochondrial genomes (Table 2; as of the end of 2023), as well as *Onchidella celtica* (Cuvier 1816) (GenBank accession number AY345048; Grande et al. 2008) as the outgroup. Sequences were aligned using MAFFT v7.450 (Katoh et al. 2002; Katoh and Standley 2013) with the FFT‐NS‐2 algorithm. A maximum likelihood tree was calculated in IQTree 2.1.2 (Minh et al. 2020; Chernomor et al. 2016) with the ModelFinder function (Kalyaanamoorthy et al. 2017) and 1000 bootstrap replicates. A Bayesian tree was calculated in MrBayes 3.2.7 (Ronquist et al. 2012) using the mixed substitution model, invgamma rate variation, and a Markov Chain Monte Carlo (MCMC) chain length of 10,000,000 generations and a subsampling frequency of every 4000 generations with the first 100,000 generations being discarded as burn‐in, four heated chains, and a chain temperature parameter of 0.2. The species tree of Romero, Pfenninger, et al. (2016) was redrawn with the set of taxa only including those also present in our phylogeny to facilitate comparisons with the mitochondrial genome trees presented here.

## 3 Results and Discussion

**FIGURE 2:**
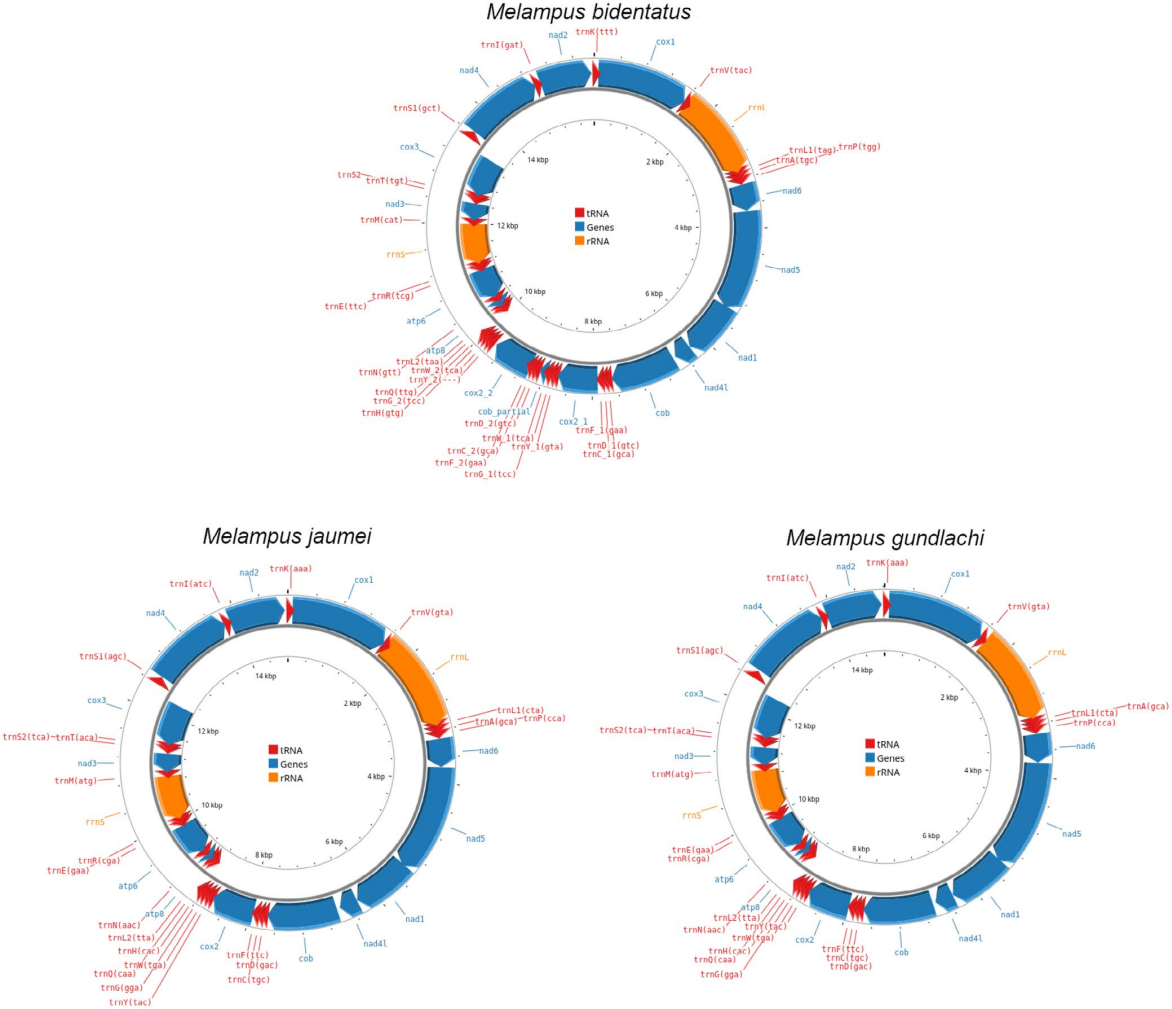
Mitochondrial genome maps of *M. bidentatus* , *M. jaumei*, and *M. gundlachi* . The maps were created on Proksee (https://proksee.ca/; Grant et al. 2023).

The mitochondrial genomes of *M. jaumei* (14,811 bp) and *M. gundlachi* (14,823 bp) are identical in gene order (Figure 2), similar in length, and contain 37 genes: 13 protein‐coding genes, 2 rRNAs, and 22 tRNAs. Twenty‐four genes are encoded on the plus strand and 13 on the minus strand. Their gene order is distinct from the conserved gene order of Euthyneura, but consistent with the gene order within the mitochondrial genome of *Melampus sincaporensis* (Yu et al. 2024). All genes encoded on the plus strand are grouped together, as are the genes encoded on the minus strand (Table S1). The gene order of the mitochondrial genome of *M. bidentatus* (15,930 bp) is largely analogous to that of the other two species, but the mitogenome has a large tandem repeat of 1201 bp encompassing parts of cytochrome B (cob), trnD, trnC, trnF, cox2, tnY, trnW, and trnG. The cox2 in the tandem repeat, as annotated in MITOS2 would start 64 bp earlier (position 9050 in the mitogenome) than the cox2 outside the tandem repeat. To avoid the resulting 45 bp overlap with the preceding trnF, the start of the tandem repeat cox2 was instead set at position 9122 (equivalent to position 9 in the cox2 from outside the tandem repeat). The tandem repeat cox2 also has a point deletion (after position 17 in the tandem repeat cox2; position 26 in the cox2 outside the tandem repeat) that would result in an early stop codon if the gene were to start at the same position as the cox2 outside the tandem repeat. The large divergence in COI sequences between the three species (Dennis and Hellberg 2010) was also present in all other mitochondrial genes in the mitochondrial genome (entire genome: 0.307–0.335, Table S3). The overall mean distance between the three cryptic *Melampus* species (0.32) is very high, especially compared to distances among all Ellobiid mitochondrial genomes with the conserved gene order (0.38, Table S4), which includes taxa from four subfamilies with a last common ancestor in the Cretaceous (Romero, Pfenninger, et al. 2016).

After machine annotation, we also compared sequences to identify a putative control region. The only non‐coding mitochondrial region shared between all Ellobiidae is a short region preceding the isoleucine tRNA sequence (trnI). Its length is similar in the three *Melampus* species ( *M. bidentatus* : 13 bp; *M. jaumei*: 11 bp; and *M. gundlachi* : 13 bp) and more variable across Ellobiidae (11–317 bp, Table S2). At least 130 of the 151 analyzed mitochondrial genomes within Euthyneura (Table S2) have a non‐coding region present in that location.

This location has been previously suggested to comprise the control region by other studies using much smaller datasets (Grande et al. 2008; White et al. 2011; Kurabayashi and Ueshima 2000) and these results (detailed in Table S2) offer further support for that.

**FIGURE 3:**
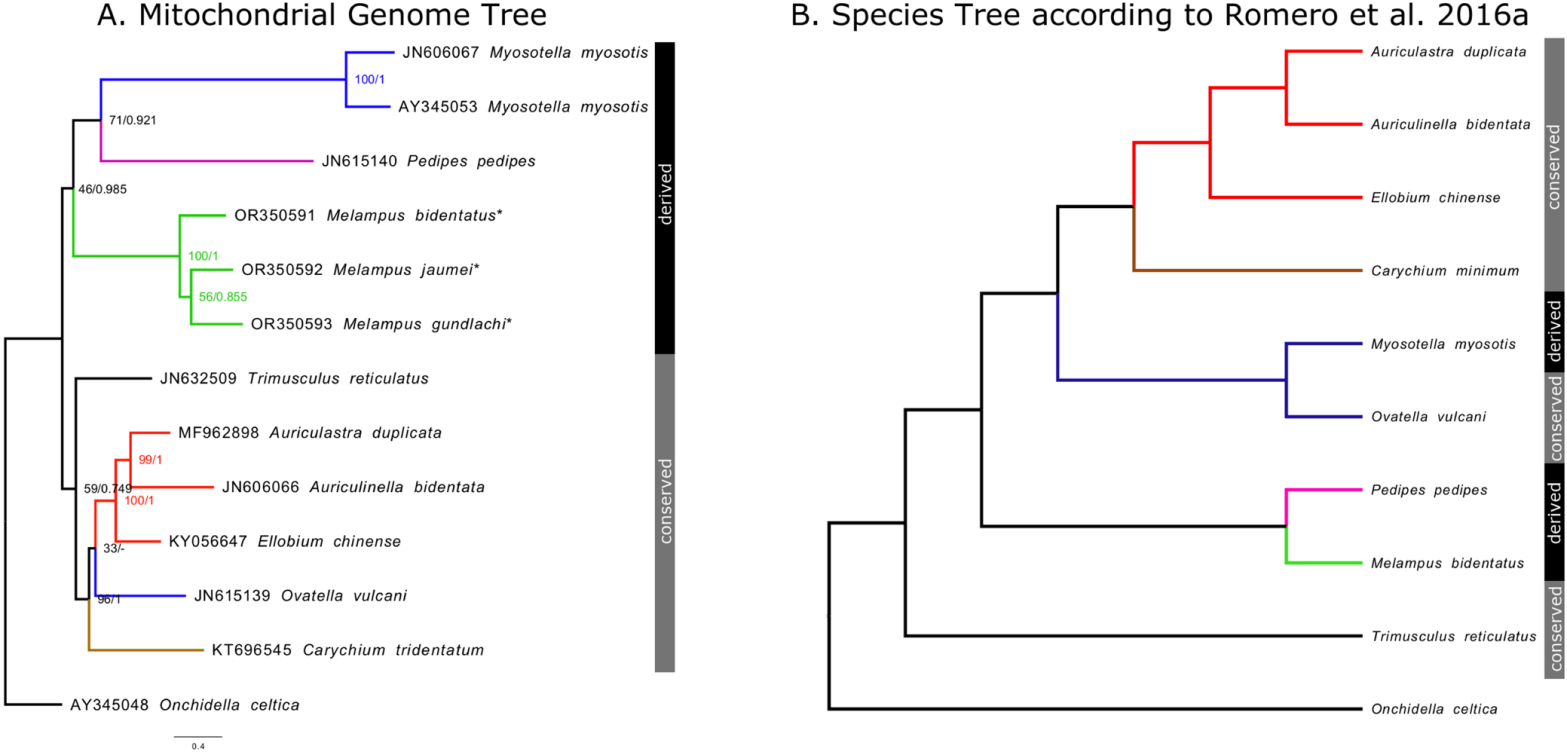
(A) Phylogenetic Tree of all published Ellobiid mitochondrial genomes. Mitochondrial genomes sequenced in this study are marked with a star (*). Node support values of both the maximum likelihood analysis (left) and the Bayesian Inference (right) are given. (B) Simplified redrawing of Romero, Pfenninger et al. (2016), Figure 1 using the same or related species as in (A). Branch color represents subfamilies: green: Melampodinae, purple: Pedipediinae, brown: Carychiinae, red: Ellobiinae, blue: Pythiinae, black: Trimusculiinae and the outgroup ( *Onchidella celtica* ). The black (conserved gene order) and gray (derived gene orders) bars to the right of the tree indicate whether a species has the conserved euthyneuran mitochondrial gene order or a derived mitochondrial gene order.

In the phylogenetic trees based on the mitochondrial protein‐coding genes (Figure 3A), the three *Melampus* species cluster with the other Ellobiid species (*Pedipes pedipes* and *Myosotella myosotis* ) with a derived mitochondrial gene order. This cluster has significant Bayesian Posterior Probabilities (BPP) (0.985) in the Bayesian tree, but a fairly poor Bootstrap Support (BS) (46%) in the maximum likelihood tree. Within that group, the clade containing *P*. *pedipes* and *M. myosotis* is decently supported in the maximum likelihood tree (BS: 71%), but not significant in the Bayesian tree (BPP: 0.921). The group with derived gene order is separated from the group containing the species with a conserved gene order, which is recovered as monophyletic in both trees, but the inclusion of *Trimusculus reticulatus* as the outgroup to the rest of the included sequences has limited support: the group as a whole was supported by the majority of the bootstrap replicates in the maximum likelihood tree (BS: 59%), but the BPP of the Bayesian tree (0.749) were not significant. In contrast, the other sequences with conserved gene order form a highly supported group (BS: 96%; BPP: 1). The position of *Ovatella vulcani* differs between the maximum likelihood (grouping outside a group consisting of *Ellobium chinense*, *Auriculastra duplicata* and *Auriculinella bidentate*; BS: 33%) and Bayesian trees (sister group of *Carychium tridentatus*; BPP: 0.797), but neither pairing has high node support values.

The group containing taxa with a conserved gene order has been recovered as monophyletic in other mitogenome phylogenies (e.g., White et al. 2011; Varney et al. 2021), albeit usually with much better node support. Within that group, Varney et al. (2021) had similar discrepancies between Bayesian and maximum likelihood trees concerning the position of *Ovatella vulcani*. The position of the specimens with derived gene orders within published mitogenome phylogenies, as well as their monophyly (White et al. 2011; Romero, Pfenninger, et al. 2016; Varney et al. 2021) varies considerably, but all taxa are consistently recovered outside the group with a conserved gene order. In contrast, the most current published Ellobiid phylogeny (Romero, Pfenninger, et al. 2016, Figure 1; redrawn here in a simplified form as Figure 3B) and previous morphological studies (Martins 1995, 1996, 2007) had specimens with derived gene orders resolved within the group with conserved gene order, with *Trimusculus reticulatus* as the sister group of all other Ellobiids. *M. myosotis* is in the previously mentioned studies instead recovered/classified within the subfamily Pythiinae together with *O*. *vulcani*, a species with conserved gene order. Romero, Pfenninger, et al. (2016) recovered *Pedipes* and *Melampus* as sister genera forming an outgroup for all Ellobiid species save *Trimusculus reticulatus* and its relatives.

Among Ellobiidae, the overall mean distance among mitochondrial genes is (except for three tRNAs) consistently higher (on average 1.57×) in taxa with a distinct gene order than among those with the conserved gene order (Table S4).

This difference in divergence between conserved and distinct gene orders might indicate that the evolutionary rates of mitochondrial genomes vary within Ellobiidae (varying evolutionary rates in Ellobiid mitochondrial genes have also been observed by Inoue et al. 2022). This can lead to false groupings of taxa with accelerated evolutionary rates in phylogenetic analyses (Liu et al. 2018), which might explain why Ellobiid mitochondrial genomes with distinct gene orders are generally recovered outside Ellobiidae in Panpulmonate or Heterobranch mitochondrial phylogenies (such as Varney et al. 2021), while included in Ellobiidae in phylogenies that include nuclear markers (Romero, Pfenninger, et al. 2016) and in morphological studies (Martins 1995, 1996, 2007).

In conclusion, the three new mitogenomes have shown that the deep divergence previously documented in a single mitochondrial gene for *Melampus* (CO1, Dennis & Hellberg) extends to all coding genes in the mitochondria. These new mitogenomes provide a resource for further studies within the genus *Melampus*, across Ellobids, and add to our broad understanding of evolutionary rates in mitochondrial genes.

## Author Contributions

**Thomas Inäbnit:** conceptualization (equal), formal analysis (equal), methodology (equal), writing – original draft (lead). **Ralph Tiedemann:** conceptualization (supporting), methodology (supporting), supervision (supporting), writing – original draft (supporting). **Alice B. Dennis:** conceptualization (equal), formal analysis (equal), funding acquisition (lead), investigation (lead), methodology (equal), supervision (equal), writing – original draft (supporting).

## Conflicts of Interest

The authors declare no conflicts of interest.

## Supporting information


Tables S1–S4

## Data Availability

Raw sequencing data has been deposited in the NCBI Sequence Read Archives ( *M. bidentatus* : PRJNA513247/SRR32554630); *M. jaumei*: PRJNA985426/SRR24965508; *M. gundlachi* : PRJNA985137/SRR24958157. Mitogenomes were deposited in GenBank under the accession numbers OR350591 ( *M. bidentatus* ), OR350592 (*M. jaumei*), and OR350593 ( *M. gundlachi* ). The individuals sequenced in this study were destructively sampled during DNA extraction, but additional individuals from the same collection or locale have been deposited at the Florida Museum of Natural History (*M. jaumei*: 3 specimens, UFID 580985‐7; *M. gundlachi* : 1 specimen, UFID 580978) and at the Royal Belgian Institute of Natural Sciences ( *M. bidentatus* : 1 specimen, INV.302.000).

## References

[ece371282-bib-0001] Allio, R. , A. Schomaker‐Bastos , J. Romiguier , F. Prosdocimi , B. Nabholz , and F. Delsuc . 2020. “MitoFinder: Efficient Automated Large‐Scale Extraction of Mitogenomic Data in Target Enrichment Phylogenomics.” Molecular Ecology Resources 20: 892–905. 10.1111/1755-0998.13160. 32243090 PMC7497042

[ece371282-bib-0002] Arab, M. A. , C. H. Zu Siederdissen , K. Tout , A. H. Sahyoun , P. F. Stadler , and M. Bernt . 2017. “Accurate Annotation of Protein‐Coding Genes in Mitochondrial Genomes.” Molecular Phylogenetics and Evolution 106: 209–216. 10.1016/j.ympev.2016.09.024. 27693569

[ece371282-bib-0003] Bernt, M. , A. Donath , F. Jühling , et al. 2013. “MITOS: Improved De Novo Metazoan Mitochondrial Genome Annotation.” Molecular Phylogenetics and Evolution 69: 313–319. 10.1016/j.ympev.2012.08.023. 22982435

[ece371282-bib-0004] Cheng, H. , G. T. Concepcion , X. Feng , H. Zhang , and H. Li . 2021. “Haplotype‐Resolved De Novo Assembly Using Phased Assembly Graphs With Hifiasm.” Nature Methods 18: 170–175. 10.1038/s41592-020-01056-5. 33526886 PMC7961889

[ece371282-bib-0005] Chernomor, O. , A. von Haeseler , and B. Q. Minh . 2016. “Terrace Aware Data Structure for Phylogenomic Inference From Supermatrices.” Systematic Biology 65: 997–1008. 10.1093/sysbio/syw037. 27121966 PMC5066062

[ece371282-bib-0006] Danecek, P. , J. K. Bonfield , J. Liddle , et al. 2021. “Twelve Years of SAMtools and BCFtools.” GigaScience 10, no. 2: giab008. 10.1093/gigascience/giab008. 33590861 PMC7931819

[ece371282-bib-0007] Dennis, A. B. , and M. E. Hellberg . 2010. “Ecological Partitioning Among Parapatric Cryptic Species.” Molecular Ecology 19: 3206–3225. 10.1111/j.1365-294X.2010.04689.x. 20618906

[ece371282-bib-0008] Donath, A. , F. Jühling , M. Al‐Arab , et al. 2019. “Improved Annotation of Protein‐Coding Genes Boundaries in Metazoan Mitochondrial Genomes.” Nucleic Acids Research 47: 10543–10552. 10.1093/nar/gkz833. 31584075 PMC6847864

[ece371282-bib-0009] Grande, C. , J. Templado , and R. Zardoya . 2008. “Evolution of Gastropod Mitochondrial Genome Arrangements.” BMC Evolutionary Biology 8: 61. 10.1186/1471-2148-8-61. 18302768 PMC2291457

[ece371282-bib-0010] Grant, J. R. , E. Enns , E. Marinier , et al. 2023. “Proksee: In‐Depth Characterization and Visualization of Bacterial Genomes.” Nucleic Acids Research 51: W484–W492. 10.1093/nar/gkad326. 37140037 PMC10320063

[ece371282-bib-0011] Inäbnit, T. , R. Tiedemann , and A. B. Dennis . 2023. “Molecular Species Delimitation and Morphometry in the *Melampus bidentatus* (Panpulmonata, Ellobiidae) Cryptic Species Complex.” Bulletin of the Society of Systematic Biologists 2, no. 2: 1–15. 10.18061/bssb.v2i2.9165.

[ece371282-bib-0012] Inoue, K. , T. Yahagi , B. Brenzinger , and Y. Kano . 2022. “Evolutionary Processes Leading to Terrestrial Invasions by Ellobiid Snails.” Spixiana Supplement 30A: 185.

[ece371282-bib-0013] Jun, J. , E. H. Choi , and H. J. Kil . 2016. “Complete Mitochondrial Genome of the Endangered Species Ellobium Chinense (Pulmonata, Ellobiidae) From Korea.” Mitochondrial DNA Part B Resources 1: 939–940. 10.1080/23802359.2016.1261609. PMC7800294 33490428

[ece371282-bib-0014] Kalyaanamoorthy, S. , B. Q. Minh , T. K. F. Wong , A. von Haeseler , and L. S. Jermiin . 2017. “ModelFinder: Fast Model Selection for Accurate Phylogenetic Estimates.” Nature Methods 14: 587–589. 10.1038/nmeth.4285. 28481363 PMC5453245

[ece371282-bib-0015] Katoh, K. , K. Misawa , K. Kuma , and T. Miyata . 2002. “MAFFT: A Novel Method for Rapid Multiple Sequence Alignment Based on Fast Fourier Transform.” Nucleic Acids Research 30: 3059–3066. 10.1093/nar/gkf436. 12136088 PMC135756

[ece371282-bib-0016] Katoh, K. , and D. M. Standley . 2013. “MAFFT Multiple Sequence Alignment Software Version 7: Improvements in Performance and Usability.” Molecular Biology and Evolution 30: 772–780. 10.1093/molbev/mst010. 23329690 PMC3603318

[ece371282-bib-0017] Kumar, S. , G. Stecher , M. Li , C. Knyaz , and K. Tamura . 2018. “MEGA X: Molecular Evolutionary Genetics Analysis Across Computing Platforms.” Molecular Biology and Evolution 35: 1547–1549. 10.1093/molbev/msy096. 29722887 PMC5967553

[ece371282-bib-0018] Kurabayashi, A. , and R. Ueshima . 2000. “Complete Sequence of the Mitochondrial DNA of the Primitive Opisthobranch Gastropod Pupa Strigosa: Systematic Implication of the Genome Organization.” Molecular Biology and Evolution 17: 266–277. 10.1093/oxfordjournals.molbev.a026306. 10677849

[ece371282-bib-0019] Li, H. 2017. “Minimap2: Fast Pairwise Alignment for Long Nucleotide Sequences.” https://arxiv.org/abs/1708.01492. 10.1093/bioinformatics/bty191 PMC6137996 29750242

[ece371282-bib-0020] Li, H. , and R. Durbin . 2009. “Fast and Accurate Short Read Alignment With Burrows‐Wheeler Transform.” Bioinformatics 25: 1754–1760. 10.1093/bioinformatics/btp324. 19451168 PMC2705234

[ece371282-bib-0021] Li, H. , and R. Durbin . 2010. “Fast and Accurate Long‐Read Alignment With Burrows–Wheeler Transform.” Bioinformatics 26: 589–595. 10.1093/bioinformatics/btp698. 20080505 PMC2828108

[ece371282-bib-0022] Liu, Y. , F. Song , P. Jiang , J. J. Wilson , W. Cai , and H. Li . 2018. “Compositional Heterogeneity in True Bug Mitochondrial Phylogenomics.” Molecular Phylogenetics and Evolution 118: 135–144. 10.1016/j.ympev.2017.09.025. 28986237

[ece371282-bib-0023] Martins, A. M. F. 1995. “Relationships Within the Ellobiidae.” In Origin and Evolutionary Radiation of the Mollusca, 285–294. Oxford University Press. 10.1093/oso/9780198549802.003.0024.

[ece371282-bib-0024] Martins, A. M. F. 1996. “Anatomy and Systematics of the Western Atlantic Ellobiidae (Gastropoda: Pulmonata).” Malacologia 37, no. 2: 163–332.

[ece371282-bib-0025] Martins, A. M. F. 2007. “Morphological and Anatomical Diversity Within the Ellobiidae (Gastropoda, Pulmonata, Archaeopulmonata).” Vita Malacologica 4: 1–28.

[ece371282-bib-0026] Minh, B. Q. , H. A. Schmidt , O. Chernomor , et al. 2020. “IQ‐TREE 2: New Models and Efficient Methods for Phylogenetic Inference in the Genomic Era.” Molecular Biology and Evolution 37: 1530–1534. 10.1093/molbev/msaa015. 32011700 PMC7182206

[ece371282-bib-0027] Romero, P. E. , M. Pfenninger , Y. Kano , and A. Klussmann‐Kolb . 2016. “Molecular Phylogeny of the Ellobiidae (Gastropoda: Panpulmonata) Supports Independent Terrestrial Invasions.” Molecular Phylogenetics and Evolution 97: 43–54. 10.1016/j.ympev.2015.12.014. 26724408

[ece371282-bib-0028] Romero, P. E. , A. M. Weigand , and M. Pfenninger . 2016. “Positive Selection on Panpulmonate Mitogenomes Provide New Clues on Adaptations to Terrestrial Life.” BMC Evolutionary Biology 16: 164. 10.1186/s12862-016-0735-8. 27549326 PMC4994307

[ece371282-bib-0029] Ronquist, F. , M. Teslenko , P. van der Mark , et al. 2012. “Mrbayes 3.2: Efficient Bayesian Phylogenetic Inference and Model Choice Across a Large Model Space.” Systematic Biology 61, no. 3: 539–542. 10.1093/sysbio/sys029. 22357727 PMC3329765

[ece371282-bib-0030] Tamura, K. , M. Nei , and S. Kumar . 2004. “Prospects for Inferring Very Large Phylogenies by Using the Neighbor‐Joining Method.” Proceedings of the National Academy of Sciences of the United States of America 101: 11030–11035. 10.1073/pnas.0404206101. 15258291 PMC491989

[ece371282-bib-0031] The Galaxy Community . 2024. “The Galaxy Platform for Accessible, Reproducible, and Collaborative Data Analyses: 2024 Update.” Nucleic Acids Research 52: W83–W94. 10.1093/nar/gkae410. 38769056 PMC11223835

[ece371282-bib-0032] Uliano‐Silva, M. , J. G. R. N. Ferreira , K. Krasheninnikova , et al. 2022. “MitoHiFi: A Python Pipeline for Mitochondrial Genome Assembly From PacBio High Fidelity Reads.” BMC Bioinformatics 24: 288. 10.1186/s12859-023-05385-y. PMC10354987 37464285

[ece371282-bib-0033] Varney, R. M. , B. Brenzinger , M. A. E. Malaquias , C. P. Meyer , M. Schrödl , and K. M. Kocot . 2021. “Assessment of Mitochondrial Genomes for Heterobranch Gastropod Phylogenetics.” BMC Ecology and Evolution 21, no. 1: 6. 10.1186/s12862-020-01728-y. 33514315 PMC7853304

[ece371282-bib-0034] White, T. R. , M. M. Conrad , R. Tseng , et al. 2011. “Ten New Complete Mitochondrial Genomes of Pulmonates (Mollusca: Gastropoda) and Their Impact on Phylogenetic Relationships.” BMC Evolutionary Biology 11: 295. 10.1186/1471-2148-11-295. 21985526 PMC3198971

[ece371282-bib-0035] Yi, C. H. , K.‐Y. Kim , T. W. Jung , et al. 2017. “Complete Sequence Analysis of the Mitochondrial Genome of Auriculastra Duplicata (Mollusca, Gastropoda, Ellobiidae).” Mitochondrial DNA Part B Resources 2: 787–788. 10.1080/23802359.2017.1398614. 33473980 PMC7800208

[ece371282-bib-0036] Yu, H. , C. R. Shin , G. Kim , B. Park , E. H. Choi , and U. W. Hwang . 2024. “The Complete Mitochondrial Genome of the Pulmonate Snail *Melampus sincaporensis* (Gastropoda: Ellobiidae) From South Korea.” Mitochondrial DNA Part B Resources 9: 1549–1553. 10.1080/23802359.2024.2429645. 39559380 PMC11571796

